# Modifications in Bone Matrix of Estrogen-Deficient Rats Treated with Intermittent PTH

**DOI:** 10.1155/2015/454162

**Published:** 2015-01-28

**Authors:** Rafael Pacheco-Costa, Jenifer Freitas Campos, Eduardo Katchburian, Valquíria Pereira de Medeiros, Helena Bonciani Nader, Keico Okino Nonaka, Lilian Irene Plotkin, Rejane Daniele Reginato

**Affiliations:** ^1^Department of Morphology and Genetics, School of Medicine, Federal University of São Paulo, 04023-900 São Paulo, SP, Brazil; ^2^Mineralized Tissue and Histology Research Laboratory, Department of Morphology and Genetics, Federal University of São Paulo School of Medicine (UNIFESP), Rua Botucatu, 740 Vila Clementino, 04023-900 São Paulo, SP, Brazil; ^3^Department of Biochemistry, School of Medicine, Federal University of São Paulo, 04044-020 São Paulo, SP, Brazil; ^4^Department of Physiological Sciences, Federal University of São Carlos, 13565-905 São Carlos, SP, Brazil; ^5^Department of Anatomy and Cell Biology, Indiana University School of Medicine, Indianapolis, IN 46202, USA; ^6^Roudebush Veterans Administration Medical Center, Indianapolis, IN 46202, USA

## Abstract

Bone matrix dictates strength, elasticity, and stiffness to the bone. Intermittent parathyroid hormone (iPTH), a bone-forming treatment, is widely used as a therapy for osteoporosis. We investigate whether low doses of intermittent PTH (1-34) change the profile of organic components in the bone matrix after 30 days of treatment. Forty 6-month-old female* Wistar* rats underwent ovariectomy and after 3 months received low doses of iPTH administered for 30 days: daily at 0.3 *µ*g/kg/day (PTH03) or 5 *µ*g/kg/day (PTH5); or 3 times per week at 0.25 *µ*g/kg/day (PTH025). After euthanasia, distal femora were processed for bone histomorphometry, histochemistry for collagen and glycosaminoglycans, biochemical quantification of sulfated glycosaminoglycans, and hyaluronan by ELISA and TUNEL staining. Whole tibiae were used to estimate the bone mineral density (BMD). Histomorphometric analysis showed that PTH5 increased cancellous bone volume by 6% over vehicle-treated rats. In addition, PTH5 and PTH03 increased cortical thickness by 21% and 20%, respectively. Tibial BMD increased in PTH5-treated rats and this group exhibited lower levels of chondroitin sulfate; on the other hand, hyaluronan expression was increased. Hormonal administration in the PTH5 group led to decreased collagen maturity. Further, TUNEL-positive osteocytes were decreased in the cortical compartment of PTH5 whereas administration of PTH025 increased the osteocyte death. Our findings suggest that daily injections of PTH at low doses alter the pattern of organic components from the bone matrix, favoring the increase of bone mass.

## 1. Introduction

Maintenance of bone mass and strength depends on the concerted actions of systemic hormones, including sex steroids and parathyroid hormone (PTH) [1]. Changes in the levels of these hormones, as in women undergoing menopause, result in decreased bone mass and increased risk of bone fractures. Similar effects are observed in aging individuals and could lead to reduced mobility and, potentially, death, which result in increased economic burden with the aging of the population [2, 3]. This justifies the need for improved treatments to prevent loss of bone mass and strength.

Intermittent PTH administration (iPTH) is the only treatment currently approved by the United States Food and Drug Administration (FDA) to increase bone mass [4]. Thus, daily injections of the hormone result in increased bone formation. Part of the anabolic effect of iPTH has been ascribed to its ability to prolong osteoblast lifespan, resulting in the accumulation of bone-forming cells with consequent increase of bone mass and mechanical resistance [5–7]. The actions of iPTH are not only restricted to formation of mineralized bone mediated by osteoblasts. Indeed, iPTH administration alters the pattern of polysaccharides present in the bone matrix. For example, expression of the hyaluronan glycosaminoglycan is increased in periosteal osteoblasts treated with iPTH [8]. In addition, altered synthesis of other glycosaminoglycans, as well as of proteoglycans, was reported in a model of mice lacking parathyroid-hormone related protein, a molecule with similar effect to PTH but with localized action [9]. Changes in the bone matrix environment can affect the fate of osteoblasts and osteoclasts [10–15] and, therefore, reduction or overexpression of bone matrix molecules could alter the result of the iPTH therapy. Indeed, the balance of glycosaminoglycans and proteoglycans is crucial for maintenance of bone, and mice with targeted-deletion of biglycan proteoglycan exhibit low bone mass, similar to osteoporosis [16].

Based on these premises, studies showing whether intermittent PTH administration alters the pattern of organic components, with focus on glycosaminoglycans and collagen are needed. Although, a recent study investigated the proteoglycans in human bone tissue after iPTH administration, that study focused on the general proteoglycan content [17]. In addition, it is known that there is a relationship between collagen cross-linking and mineralization of bone matrix in monkeys and humans treated with low doses of iPTH at long term, indicating that iPTH influences the maturity of collagen fibers [18, 19].

Thus, we aimed to investigate the effects of low doses of iPTH at short term on the main organic bone matrix constituents in rats in which osteopenia was induced by sex steroid removal through ovariectomy. We found out that intermittent administration of PTH alters the pattern of organic components from the bone matrix, potentially favoring bone formation.

## 2. Material and Methods

### 2.1. Animals and Treatment

Forty 6-month-old female* Wistar* rats (260 to 270 g) underwent bilateral ovariectomy (OVX) under intraperitoneal anesthesia with ketamine (40 mg/kg) and xylazine (20 mg/kg) to induce osteopenia within 3 months after surgery [20–22]. Rats were randomly assigned to four groups (*n* = 10/group). The animals received saline injections subcutaneously (abbreviated as OVX) or PTH at 0.3 *μ*g/kg/day (abbreviated as PTH03) or 5 *μ*g/kg/day (abbreviated as PTH5) 7 times a week for 30 consecutive days, totalizing 30 injections. An additional group of animals received 0.25 *μ*g/kg/3 times a week (abbreviated as PTH025), only on Mondays, Wednesdays, and Fridays for 30 days, totalizing 12 injections. Human PTH (1-34) (Calbiochem, Darmstadt, Germany) was dissolved in saline before administration. Rats were euthanized 24 hours after last injection by anesthesia overdose and then distal femora and whole tibiae were collected. All protocols involving rats were approved by the Institutional Animal Care and Use Committee of Federal University of São Paulo (UNIFESP, process number 0643/08).

### 2.2. Vaginal Smears Collection

**Figure 1:**
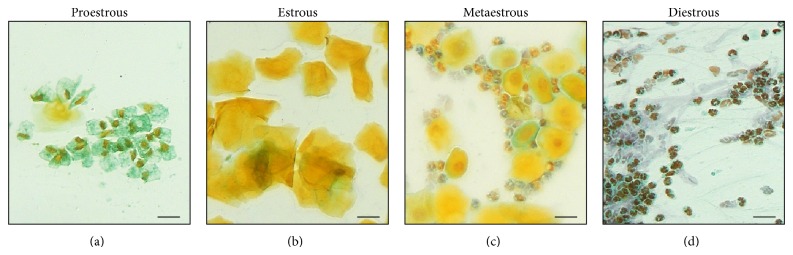
Characterization of estrous cycle in ovariectomized mice in relation to proportion of cells: (a) proestrous, showing a predominance of polynucleated and rounded cells; (b) estrous, showing decreased amount of epithelial cells and predominance of anucleated cornified cells; (c) metaestrous, containing the same proportion of leukocytes and nucleated epithelial cells, and (d) diestrous (in this case, anestrous, without permanent hormonal stimulus), containing a predominance of leukocytes in the smear. The scale bar represents 10 *μ*m.

Vaginal smears were collected from all rats for 4 consecutive days in order to confirm the success of the ovariectomy procedure by analyzing the periodicity for the estrous cycle 21 days after removal of the ovaries. For this, a cotton swab was dampened with saline and then carefully introduced into a vaginal cavity and slightly rotated. The secretion containing cells was placed on glass slides and fixed in ether and 95% ethanol (1 : 1) for 20 min and evidenced by Shorr staining [23]. As inclusion criteria, only rats that were at diestrus (or anestrous) for at least three consecutive days were used in this study. In the current study, all 40 rats ovariectomized were arrested at diestrus phase (Figure 1).

### 2.3. Histological Preparations

Distal femora were fixed in 4% formaldehyde (freshly derived from paraformaldehyde) buffered at pH 7.2 with 0.1 M sodium phosphate, at room temperature for 4 days. Bones were subsequently decalcified in 25% formic acid for 30 days, replacing once a week the decalcification solution. Samples were then dehydrated in graded concentrations of ethanol, embedded in paraffin, and consecutive 5 *μ*m thick sections were processed for histomorphometry, histochemistry, and TUNEL staining, as described below.

### 2.4. Bone Histomorphometry

**Figure 2:**
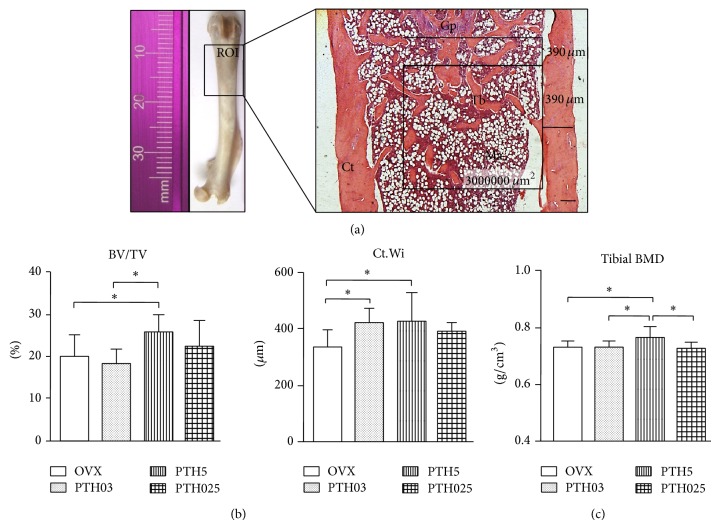
Increased bone mass in rats treated with low doses of intermittent PTH. (a) Photo from whole femur. The black box indicates the region of interest (ROI) in the distal femora that histomorphometry, histochemistry, TUNEL assay, and GAGs quantification were performed. Observe a light micrograph from bone section stained with hematoxylin and eosin (H&E) showing the region of interest for histomorphometric analysis (black square). An area of 3 mm^2^ was evaluated at 390 *μ*m below the lowest point of the growth plate and 390 *μ*m from the outer cortical surface. To measure cortical thickness, the average from three cortical thicknesses per section was calculated in the metaphyseal region, using the mean of three measurements from the same box used to measure the cancellous area. Ct: cortical; Ma: bone marrow; Tb: trabecula; Gp: growth plate. Scale bar represents 200 *μ*m. (b) Cancellous bone volume (BV/TV) and cortical thickness (Ct.Wi) were measured by histomorphometry in the ROI of estrogen-deficient rats treated with vehicle or at indicated doses of iPTH. (c) Tibial BMD was measured in whole tibia by Archimedes principle. Data are expressed as mean ± SD. *N* = 9–11. ^*^ *P* < 0.05 by one-way ANOVA.

Bone sections were stained with hematoxylin and eosin (H&E) to visualize the bone morphology and submitted to histomorphometric analysis. In order to define the region of interest for cancellous bone, an area of 3 mm^2^ was evaluated 390 *μ*m below the lowest point of the growth plate (to exclude the primary spongiosa) and 390 *μ*m from the outer cortical surface (25x magnification) (Figure 2(a)). To measure cortical thickness, the average from three measurements per section was calculated in the metaphyseal region. Histomorphometric procedures were carried out using a semiautomatic image analysis system (AxioVision Rel. 4.6., Carl Zeiss, Germany). At least five consecutive bone sections from each animal were examined. The histomorphometric indices were reported according to the standardized nomenclature recommended by the American Society of Bone and Mineral Research [24]. The following parameters were analyzed: cancellous bone volume (BV/TV, %) and cortical thickness (Ct.Wi, *μ*m).

### 2.5. Bone Mineral Density by Archimedes Principle

Tibiae were cleaned from the adhered tissues and stored in 0.9% saline at −20°C, until used. For bone mineral density measurements, tibiae were placed in a desiccator for 24 hours and then immersed in distilled H_2_O to obtain the immersed weight. Bones were then dehydrated at 100°C for 24 hours, followed by incineration at 800°C for additional 24 hours, before obtaining dry and mineral weight [21, 22]. Estimation for bone mineral density was performed by using the following formula: Bone mineral density (BMD) = mineral weight/bone volume. Bone volume = weight after 24 h inside a desiccator − immersed weight/water density.

### 2.6. Histochemistry for Glycosaminoglycans

Bone sections were stained by the alcian blue method in order to identify glycosaminoglycans in the distal femur from 390 *μ*m below from the lowest point of the growth plate, in the same area used to histomorphometry analyses. Sulfated GAGs were detected at pH 0.5 and all glycosaminoglycans, including hyaluronan at pH 2.5 [21, 22]. Briefly, sections were deparaffinized, rehydrated, and incubated in either 0.5 M HCl or 3% acetic acid for 2 min before staining with alcian blue at pH 0.5 or pH 2.5, respectively, for 40 minutes. Sections were dehydrated, cleaned, and mounted in entellan [21, 22]. Glycosaminoglycans were identified and quantified by measuring the distribution and intensity of the bluish staining (ImageLab 2000 system, Diracom, Brazil).

### 2.7. Histochemistry for Collagen Fibers and Hyaluronan

Bone sections were stained using a modified picrosirius red technique previously described, and the birefringence of collagen fibers was evaluated in the distal femur from 390 *μ*m below the lowest point of the growth plate [21, 22, 25]. Sections were deparaffinized, rehydrated, and incubated with 0.2% aqueous phosphomolybdic acid for 10 minutes to render the cytoplasm colorless and then stained with 0.1% solution of sirius red diluted in saturated aqueous picric acid for 90 minutes. Next, sections were rinsed with 0.01 M HCl for 2 minutes, dehydrated, cleaned, and mounted. One section from each animal was captured with a microscope (Axioskop 40, Carl Zeiss, Germany) equipped with polarizating filters using a digital camera (AxioCam MRc 5, Carl Zeiss, Germany).

To detect the hyaluronan expression, distal femur bone sections were deparaffinized, rehydrated, and incubated with 3% hydrogen peroxide to block endogenous peroxidase activity, followed by incubation with 1% BSA for 40 min to block nonspecific binding sites. Next, sections were incubated overnight with biotinylated hyaluronan-binding protein, produced by Nader et al. [26, 27]. After rinsing with PBS, sections were incubated with streptavidin-peroxidase solution (Santa Cruz Biotechnology, Santa Cruz, CA) for 1 hour at room temperature in a dark chamber. Samples were developed using DAB substrate-chromogen system (Dako North America Inc., Carpinteria, CA) for up to 5 min. Sections were then rinsed and counterstained with Carrazi's hematoxylin. The final product of the reaction presented a brownish color, corresponding to the bindings of endogenous hyaluronan to the probe [26, 28].

### 2.8. TUNEL Assay for Cell Death

For detection of DNA breaks the Apop-Tag Peroxidase* In Situ *Apoptosis Detection Kit (Chemicon Internacional, Temecula, CA) was used as previously described [29]. TUNEL-positive hypertrophic chondrocytes were observed at the growth plate, serving as an internal positive control for each section. Negative controls were prepared by replacing TdT enzyme by distilled water. Three animals/group and two sections/rat were scored for determination of the number of TUNEL-positive osteocytes.

### 2.9. Quantification of Sulfated Glycosaminoglycans (GAGs) and Hyaluronan

In a preliminary study, we compared 10% EDTA and 25% formic acid, as decalcification solutions, in order to evaluate which one showed better efficiency for releasing smaller amount of glycosaminoglycans, at different time points. This standardization was crucial to not overestimate or subestimate the concentration of glycosaminoglycan expressed on bone tissue, indicating that 25% acid formic was more efficient in 3 days, allowing the maceration and releasing a low amount of glycosaminoglycans. After removing the cartilage from the articular cartilage up to growth plate from the distal femora, the samples were incubated in phosphate buffered solution containing protease inhibitors for 3 hours, followed by decalcification in 25% formic acid for 3 days. Five samples per group were then macerated with liquid nitrogen, dried, and incubated for 18 h at 60°C in 0.1 M phosphate buffer-cysteine, pH 6.5 containing 2 mg/mL papain, and 0.02 M ethylenediaminetetraacetic acid (EDTA) to release the GAGs (Calbiochem, Darmstadt, Germany). Ninety percent trichloroacetic acid was then added to a final concentration of 10% to precipitate the proteins and nucleic acids at 4°C, followed by two volumes of cold methanol with shaking to precipitate the GAGs and incubated at −20°C for 24 h. The supernatant was then discarded and the material was dried at room temperature. The pellet dissolved in water was applied to a 0.55% agarose gel in 0.05 M 1,3-diaminopropane acetate buffer (pH 9.0) [30]. The gel was fixed with 0.1%* N*-cetyl-*N*,*N*,*N*-trimethylammonium bromide solution for 2 hours, dried, and stained with 0.1% toluidine blue in acetic acid/ethanol. The intensity of the bands was measured using a QuickScan 2000 densitometer (Helena Laboratories Corp., Beaumont, TX) [21, 28]. Hyaluronan concentration was determined using an ELISA-like assay [27]. Briefly, standard concentrations (0–500 *μ*g/L) of hyaluronan from umbilical cord (Sigma Aldrich) and aliquots from bones obtained after proteolysis were diluted blocking buffer, and 100 *μ*L from each sample was added in triplicate to 96-well plates coated with hyaluronan-binding protein. After incubation for 18 hours at 4°C, the plates were washed 3 times with washing buffer containing 0.05 M Tris-HCl, 0.15 M NaCl, 0.05% Tween 20, 0.02 mM EDTA III, 7.7 mM sodium azide, and pH 7.75 and followed by addition of 100 *μ*L of biotinylated hyaluronan-binding protein (1 mg/mL) to each well. Plates were maintained shaking for 2 hours at room temperature and then washed 6 times with washing buffer before adding 100 *μ*L of europium-labeled streptavidin. After 30 minutes of incubation at room temperature, the plates were washed 6 times. After that, 200 *μ*L of enhancement solution (Perkin-Elmer Life Sciences) was added and then shaken for 10 minutes to release the europium-bound streptavidin. The released europium was read at 340 and 630 nm (excitation and emission filters, resp.) [26, 31].

### 2.10. Statistical Analysis

The data were analyzed by using GraphPad Prism (GraphPad Software Inc., La Jolla, CA). The groups were compared using one-way ANOVA followed by Tukey's multiple comparison test to evaluate differences among groups. Significance was established at *P* < 0.05. All numerical values were reported as mean ± standard deviation (SD).

## 3. Results

### 3.1. Low Dose of Intermittent PTH Increases Bone Mass in Cancellous and Cortical Compartment

Analysis in the femoral bone microarchitecture from rats treated with low doses of intermittent PTH (1-34) for one month showed an increase of 6% in the cancellous bone volume (BV/TV) of PTH5 compared to the OVX group and of 8% compared to the PTH03 group (Figure 2(b)). On the other hand, there were no statistical differences in BV/TV between PTH03 and PTH025 compared to the OVX group. In addition, there is also no difference in PTH025 when compared to PTH5 and PTH03 group. Cortical thickness (Ct.Wi) increased by 21% in PTH03 and 20% in PTH5 groups compared to OVX, while the effect of PTH025 did not reach statistical significance. Furthermore, tibial BMD increased only in PTH5 group and was significantly higher compared to all other groups (Figure 2(c)).

### 3.2. Glycosaminoglycans Profile Is Altered after Treatment with iPTH

**Figure 3:**
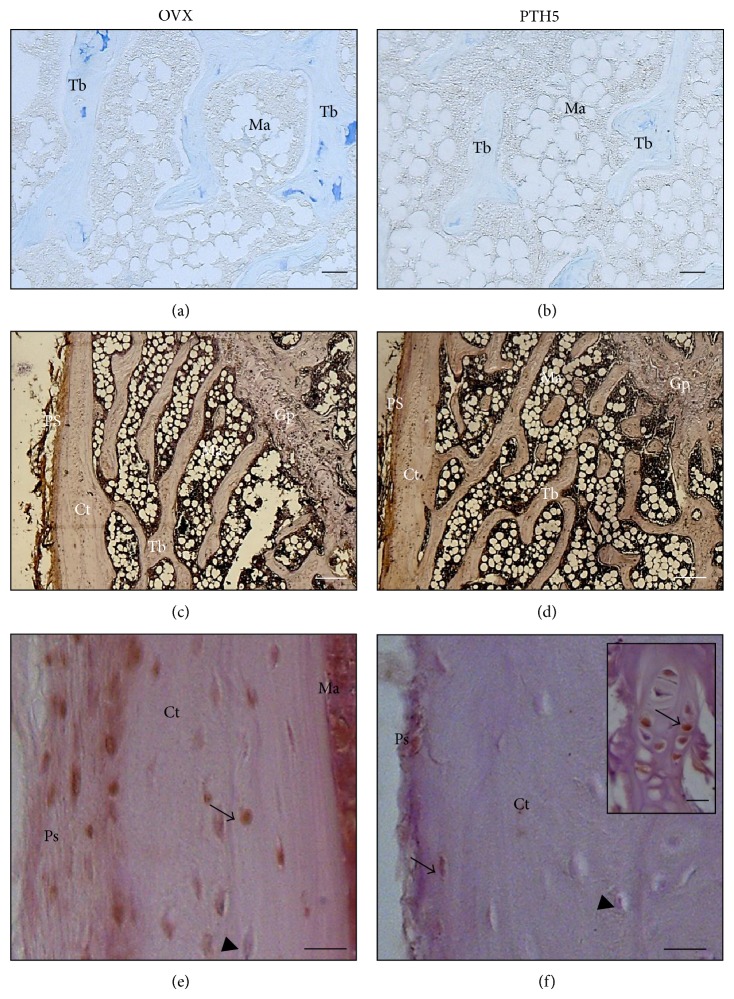
(a and b) Modifications in the glycosaminoglycans after iPTH administration. Representative images of 10 sections/group under bright field micrographs. Sections of distal femora were stained by alcian blue, specific for sulfated GAGs (at pH 0.5). (a) OVX rats exhibiting more expression of sulfated GAGs compared with (b) PTH5 group. Scale bars represent 10 *μ*m. (c and d) Representatives light micrographs of femoral sections stained for hyaluronan. Bone sections from (c) OVX rats exhibiting slightly less expression of hyaluronan expression (brownish color) compared with (d) PTH5-treated rats. Scale bars represent 200 *μ*m. Ps: periosteum; Ct: cortical; Ma: bone marrow; Tb: trabecula; Gp: growth plate. (e and f) Representatives light micrographs of distal femora from rats stained by TUNEL for the detection of DNA breaks. (e) Femoral bone sections from OVX rats exhibit more TUNEL-positive when compared with (f) bone sections from PTH5 group. Arrowhead points at TUNEL-negative osteocytes and the arrow points at TUNEL-positive osteocytes. Inset shows TUNEL-positive hypertrophic chondrocytes (arrows) at the growth plate, serving as an internal positive control for each section. *N* = 3 sections/group. Scale bars represent 10 *μ*m. The number of TUNEL-positive osteocytes is expressed as mean ± SD. Ct: cortical; Ma: bone marrow; Tb: trabecula.

We found a decrease in sulfated and carboxylated GAGs at pH 2.5 and also in sulfated GAGs only at pH 0.5 of PTH5 and PTH025 groups when compared to the OVX group (Table 1). In addition, bone sections from the OVX group evidenced the highest predominance of GAG at pH 0.5 (Figure 3(a), blue staining) compared to the lowest predominance in PTH5 rats (Figure 3(b)).

A similar electrophoretic mobility of chondroitin sulfate was found in the distal femur lysates, whereas dermatan sulfate and heparan sulfate were not detected (not shown). Quantification of chondroitin sulfate showed a decreased in all iPTH-injected groups compared to control but reached significance only in PTH5 group, with an approximately 41% reduction. Moreover, hyaluronan quantification by ELISA showed an increase only in the PTH5 group, compared to control (Table 1). Consistent with that, the expression of hyaluronan in femoral bone sections was slightly stronger in the PTH5 group (Figure 3(d)), compared to the OVX group (Figure 3(c)).

### 3.3. Behavior of Collagen Birefringence Changed in iPTH-Treated Rats

**Figure 4:**
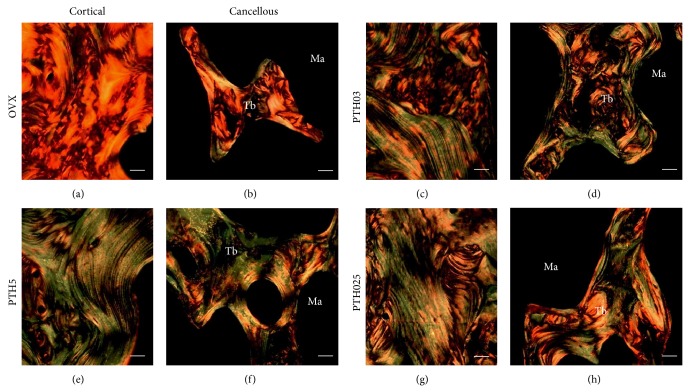
Altered collagen pattern in iPTH-treated rats. Representatives light micrographs from 10 sections of distal femur/group, evidenced by picrosirius under polarized light. Cortical and cancellous compartments bone of the groups (a, b) OVX, (c, d) PTH03, (e, f) PTH5, and (g, h) PTH025. Scale bars represent 200 *μ*m. Ma: bone marrow; Tb: trabecula.

In order to examine the effect of iPTH on the pattern of collagen in femoral bone sections, we used the picrosirius-polarized light method. Bones from the OVX group exhibited a predominance of reddish birefringence in the cortical and cancellous compartment (Figures 4(a) and 4(b)) in comparison with reduced reddish birefringence and increased greenish birefringence in all iPTH-treated groups (Figures 4(c)–4(h)), in particular, in the bone sections from the PTH5 group (Figures 4(e) and 4(f)), suggesting an altered pattern of collagen induced by iPTH administration, an indicative of collagen immaturity.

### 3.4. Decreased Osteocytes Death in the PTH-Injected Groups

To investigate whether inhibition of cell death induced by iPTH is associated with bone matrix modifications, we quantified the prevalence of osteocytes death in cortical bone stained using the TUNEL assay. In comparison to the OVX group (Figure 3(e)), we detected a decrease in TUNEL-positive osteocytes in the cortical compartment of PTH03 and PTH5 group; however, these changes did not reach the significance, but PTH5 exhibited a tendency toward increase (Table 2 and Figure 3(f)). On the other hand, PTH025 increased significantly the number of TUNEL-positive osteocytes by 50% when compared to OVX group (Table 2).

## 4. Discussion

We show herein that low doses of intermittent PTH are able to increase bone mass in estrogen-deficient rats and that this effect is accompanied by modifications in the profile of secreted components in the bone matrix. Daily treatment with 5 *μ*g/kg/day of iPTH for one month increased both cancellous and cortical bone mass. However, administration of 0.3 *μ*g/kg/day every day only increased cortical bone thickness. These results suggest that cancellous and cortical bone display a different sensitivity to low doses of iPTH. Indeed, Iida-Klein et al. [32] showed that long bones responded to iPTH already between 1-2 weeks, whereas the increase in vertebral bone was only detected after 7 weeks in mice treated with a dose of 40 *μ*g/kg/day. Other studies also reported that the hormone has skeletal site-specific action, promoting the cortical increase through a mechanism that involves increased in both bone formation and resorption in the cortical bone [33–36].

Consistent with the anabolic action of iPTH, tibial BMD increased in rats receiving a dose of 5 *μ*g/kg/day. Several authors have reported that doses of iPTH from 20 up to 100 *μ*g/kg also have anabolic effects, increasing bone mass and reducing risk for fractures [5, 7, 32, 37]. We now report the effectiveness of 0.3 *μ*g/kg and 5 *μ*g/kg doses, administrated 7 days a week by 30 days, in ovariectomy-induced bone loss, an experimental model of low bone density in rats [20–22, 38], thus suggesting that daily injections of PTH increase cancellous and cortical bone in very low doses. Instead, administration of 0.25 *μ*g/kg only 3 times a week had little, if any, effect on bone mass, showing that short time applied in this study or the low cumulative dose was not enough to increase cancellous and/or cortical bone mass. Similarly, a previous study by Turner et al. [39] showed that a dose of 1 *μ*g/kg/day PTH prevented the bone loss induced by unloading but did not increase BMD after 2 weeks of therapy.

Although other studies also have reported the effectiveness of low doses of iPTH, the attention was limited to the effect of the hormone on BMD. In our study, we focused particularly on modifications in the pattern of bone matrix organic components, which might drive cell fate and consequently act in favor of formation and/or resorption, depending on molecules induced by iPTH administration. Administration of low doses of iPTH might favor the detection of changes in the bone matrix at the end of 30-day treatment, since higher doses could accelerate the bone matrix modifications, missing the opportunity to detect the changes in the components of the bone matrix.

The current clinical dose of iPTH approved to severe osteoporotic patients is 20 *μ*g, independently of weight; however, considering that patients weigh between 60 and 70 kg, the direct final dose injected is ~0.3 *μ*g/kg (PTH03) [40]. Thus, we created the first iPTH-treated group (PTH03), receiving this dose. The other iPTH-treated group (PTH025), was created in an attempt to detect possible changes in bone components, the same changes expect to be found in PTH03, but in rats receiving PTH only 3 times a week. We also created a group receiving 5 *μ*g/kg/day, approximately 17 times more PTH than PTH03, but still considered a low dose, in order to enhance the changes in bone tissue constituents.

Collagen is the most abundant component of organic bone matrix [41], and altered collagen is associated with bone fragility in animals and humans [42–46]. In the current study, we evaluated the collagen birefringence behavior by picrosirius red-polarization, a well-known method to assess collagen maturation [25, 47]. We observed a dramatic increase in greenish birefringence in all iPTH-injected groups, especially in that group receiving the highest dose (PTH5). Immature and mature collagen fibrils of bone are differentiated by their colors under polarized light [48]. Against a black background, thick fibers are mainly type I mature collagen, consequently present intense birefringence of yellow to red color, while thin fibrils formed mainly by type I immature collagen (including procollagen, intermediaries, and even altered collagen) and display a weak birefringence of greenish color [48–50]. Since the PTH5 group exhibited the highest predominance of thin fibers, we propose that in this group the hormone stimulates new bone tissue deposition, initially reflecting on immature type I collagen content. In addition, we cannot rule out the possibility that rats treated with PTH5 are facing a higher rate of collagen fibers remodeling at the end of 30-day treatment, when compared to the OVX group. Collagen birefringence changes also can be attributed to orientation of the fibers [51]. In addition, greenish birefringence is also associated with accumulation of type III collagen [52]. We propose that in our current study, this birefringence is associated with changes in morphology rather than with increased synthesis of type III collagen. Consistent with this notion, the expression of the gene encoding the type III collagen in rats is not altered when PTH is administered in the intermittent fashion [53].

Studies have associated changes in collagen morphology and thickness with the presence of glycosaminoglycans, in particular, those containing sulfated residues [21, 22, 54, 55]. Moreover, sulfated GAGs have important roles in the initial process of mineralization [12, 55]. Based on this idea, we investigated the effect of the treatments on the levels of chondroitin sulfate, the most abundant sulfated GAG in bone tissue [56]. Expression of sulfated GAG is reduced in the bone sections from PTH5 and PTH025 group assessed by histochemistry, but the results obtained with the biochemical quantification revealed that only rats receiving PTH5 exhibited a reduction in chondroitin sulfate. The difference on the findings between histochemistry for GAGs and biochemical analysis in the PTH025 group might result from the fact that while the histochemistry method detects other sulfated GAGs on the bone sections, like dermatan and heparan, biochemistry analysis detects exclusively chondroitin. In addition, differences in the sensitivity of the methods could also explain this discrepancy. Nevertheless, it has been shown that fusion and thickening of collagen fibrils are triggered by removal of sulfated GAGs [54, 55, 57]. Supporting this idea, in a deletion study using biglycan- and decorin-deficient mice chondroitin sulfate-rich proteoglycans showed altered morphology such as decreased diameter and size of collagen fibrils [54]. Thus, the removal of chondroitin sulfate might have led to the changes observed in the birefringence of collagen fibers. The changes in collagen birefringence observed in our study could be at least partially explained by a reduction in sulfated GAGs.

We found that hyaluronan expression is increased in bone lysates of rats treated with iPTH, mainly with the highest dose. It has been reported the accumulation of hyaluronan in periosteal area of long bones and increased expression in cells of the osteoblastic lineage after treatment with iPTH [8, 58]. However, since it was not our intention to detect hyaluronan expression in the periosteum, we scrapped off this layer from the bone when preparing it. Nevertheless, we detected small areas of periosteum exhibiting increased expression for hyaluronan, similar to what was reported by Midura and coworkers [8]. Further studies are needed to determine the role of the increase in hyaluronan induced by iPTH on the periosteal surface.

It has been proposed that osteoporosis and other age-related diseases are caused, in part, by apoptosis of bone-forming cells [59–61]. iPTH is thought to improve osteoporosis by stimulating bone matrix production and by suppressing osteoblast and osteocyte apoptosis [59, 62]. Here we show that the hormone also reduced osteocyte death in cortical bone sections from the PTH03 and PTH5 groups. However, we found a dramatic increase in osteocyte apoptosis in the rats treated with PTH025. Although the reason for this increase is not known, we can speculate that is due to the fact that the hormone is not administered daily and the cell death is an initial event during iPTH administration that is delayed in this group compared to other iPTH-treated groups and can be detected at the end of 30-day treatment. Consistent with this possibility, a study showed that daily injections with a higher dose of PTH (80 *μ*g/kg/day) resulted in an increase in apoptotic osteocytes in cancellous bone after 7 days of treatment in rats and that the percentage of apoptotic cells return to basal levels 28 days after initiating hormone administration [63]. Taking into account that the iPTH dose used in the above study was much higher than those administrated by us, it is reasonable to propose that in the group injected with the very low concentration of hormone and only 3 times a week (PTH025) it still is not returned to the basal levels (as observed after 28 days by Stanislaus et al. [63]) at the end of the treatment period (30 days). These pieces of evidence suggest that the low iPTH doses at short time could induce transient increase in osteocyte death that will be followed by a reversion of the effect.

Terminal deoxynucleotidyl transferase (TdT) mediated dUTP nick-end-labeling (TUNEL) is widely considered as a “gold standard” assay for assessment of apoptosis. However, there are exceptions for TUNEL specificity [6, 60], since this assay can identify nucleotide excision repair (NER) [64, 65]. NER involves the creation of DNA breaks through an endonuclease driven excision of damage nucleotides creating 3′OH groups [65, 66]. TdT, the enzyme used to label nicked DNA in the TUNEL reaction, recognizes 3′OH groups at nicked ends in DNA identifying DNA breaks in fixed cells [6, 65]. In our study some TUNEL-positives osteocytes could be undergoing DNA repair. Schnoke et al. [6] hypothesized that PTH might suppress osteoblast apoptosis by enhancing DNA repair and they showed that the attachment of nucleotides to the 3′OH ends of fixed DNA via the TUNEL reaction in PTH-treated nuclei was more likely generated by NER-mediated nicking and not apoptotic cleavage. Whether the TUNEL-positive cells are indeed apoptotic or whether DNA repair is taking place will be the subject of future studies.

The signals that trigger bone resorption are not completely understood. One important event in the regulation of remodeling seems to be apoptosis of osteocytes. The death osteocytes release chemotactic signals that attract osteoclasts, triggering localized bone resorption [67]. During the bone remodeling process, the matrix is resorbed before the new tissue is produced. If, as proposed, an early effect of PTH administration is to induce osteocyte death, this will be followed by localized bone resorption and the consequent remodeling of the matrix. Our results suggest that in the PTH5 group, at the dose of 5 *μ*g/kg/day, the osteocyte death phase has most likely already occurred, and the stage detected here is associated with the strong remodeling of the matrix, demonstrated by collagen fiber turnover, as indicated by the greenish birefringence, low concentration of sulfated GAGs, and high expression of hyaluronan.

One limitation of our study is that we did not use a group of rats without undergoing ovariectomy (sham group). However, the initial propose of this study was to compare whether the low doses of iPTH changes the profile of organic bone matrix components after 30 days of treatment in rats with osteopenia. Although we cannot evaluate if bone matrix components are altered compared to sham controls, we can analyze the changes in bone matrix induced by iPTH administration and compare it with the OVX model.

In summary, we show herein that low dose of daily iPTH administration results in changes in organic component of bone matrix, such as in glycosaminoglycans and collagen. These events, together with increased bone mass and reduction in osteocyte cell death could contribute to increased bone strength in individuals receiving intermittent administration of the drug.

## Figures and Tables

**Table 1:** Histochemical and biochemical quantification of glycosaminoglycans.

	Histochemical analysis		Biochemical analysis	
	General GAGs pH 2.5 (%)	Sulfated GAGs pH 0.5 (%)	Chondroitin sulfate (*µ*g/mg tissue)	Hyaluronan (*µ*g/g tissue)
OVX	3.68 ± 2.21	2.98 ± 1.54	0.90 ± 0.08	29.18 ± 13.08
PTH03	3.02 ± 1.97	1.81 ± 0.99	0.67 ± 0.11	33.24 ± 12.16
PTH5	1.40 ± 0.83^*^	1.24 ± 1.55^*^	0.53 ± 0.08^*^	58.71 ± 13.48^*^
PTH025	1.72 ± 1.26^*^	1.38 ± 1.28^*^	0.57 ± 0.11	33.66 ± 13.16

Data are expressed as mean ± SD. *P* < 0.05; ^*^versus OVX.

**Table 2:** Quantification of TUNEL—positive osteocytes in the cortical compartment.

TUNEL, positive osteocytes (total number/section)	
OVX	27 ± 16
PTH03	18 ± 1
PTH5	5 ± 5
PTH025	56 ± 11^*^

Data are expressed as mean ± SD. *P* < 0.05; ^*^versus OVX.
